# Real world experience with cyclin dependent kinase inhibitors in metastatic breast cancer in India

**DOI:** 10.1371/journal.pone.0349196

**Published:** 2026-05-15

**Authors:** Anindya Mukherjee, Pankaj Goyal, Rupal Tripathi, Sumit Goyal, Ullas Batra, Vineet Talwar, Varun Goel, Arpit Jain, Shekhar Saha, Sneha Bothra, Chaturbhuj Agrawal, Swati Chugh, Dinesh Chandra Doval

**Affiliations:** 1 Department of Medical Oncology, Rajiv Gandhi Cancer Institute & Research Centre, Delhi, India; 2 Department of Research, Rajiv Gandhi Cancer Institute & Research Centre, Delhi, India; King Faisal Specialist Hospital and Research Center, SAUDI ARABIA

## Abstract

**Background:**

The present study evaluated the real-world experience with cyclin dependent kinase 4/6 inhibitors palbociclib and ribociclib in hormone receptor-positive/ human epidermal growth factor receptor 2-negative (HR + /HER2-) metastatic breast cancer.

**Method:**

This study was conducted on the HR + /HER2- metastatic breast cancer patients who received palbociclib/ ribociclib in the first line or recurrent setting between January 2021 and January 2023 along with endocrine therapy. Data was retrieved from the Hospital medical records.

**Results:**

In the patients receiving palbociclib, a better median progression free survival (PFS) was observed in patients with Eastern Cooperative Oncology Group (ECOG) performance status (PS) 1 (27.3 months,p-value <0.0001), *de novo* disease (17.5 months,p-value 0.0155), first line hormonal therapy (22.5 months,p-value <0.0001) and no prior chemotherapy (20.3 months,p-value 0.0001). Similarly, in the patients receiving ribociclib, a better median PFS was observed in patients with ECOG PS 1 (22.2 months,p-value <0.0001), *de novo* disease (21.7 months,p-value 0.0042), first-line hormonal therapy (22.5 months,p-value <0.0001) and no prior chemotherapy (21.7 months,p-value <0.0001). On multivariate analysis, ECOG PS, line of hormone therapy and prior chemotherapy showed significance (p-values <0.0001, < 0.0001 & 0.0269, respectively).

**Conclusion:**

The study shows real-world experience with cyclin dependent kinase 4/6 inhibitors palbociclib and ribociclib in metastatic breast cancer in India.

## Introduction

Hormone receptor positive/ human epidermal growth factor receptor 2 negative (HR + /HER2-) breast cancer is the most common molecular subtype, accounting for 68% of all breast cancers and having the highest incidence rate of 87.4 new cases per 100,000 women based on Surveillance, Epidemiology, and End Results (SEER) data of 2015–2019 [1]. Although HR + /HER2- subset has the best prognosis amongst all molecular subtypes of breast cancer, most patients ultimately progress on treatment with traditional endocrine therapies [2].

Various targeted agents have been tried over the years and CDK inhibitors (CDKi) are the latest class of drugs in the armamentarium against HR+ /HER2- advanced/ metastatic breast cancer (ABC/MBC). The CDK4/6 inhibitors: palbociclib, ribociclib, and abemaciclib received FDA-approval between 2015–2017 for the treatment of HR+ /HER2− MBC. Updated results of PALOMA, MONALEESA and MONARCH trials have demonstrated significant benefits in Progression-free survival (PFS) with these drugs over endocrine therapy (ET) alone in both first and second-line treatment of breast cancer [3–5]. Significant overall survival (OS) benefit was also demonstrated by these agents except palbociclib and abemaciclib in first line therapy in postmenopausal women [6–15]. Several systematic reviews and meta-analyses conducted on studies on CDKi in HR + /HER2- MBC between 2018–2021 have shown a significant (pooled) OS benefit with a strikingly similar HR of 0.76 (95% CI 0.68–0.85) with respect to endocrine therapy alone [16–18] whereas others have also reported a significant (pooled) PFS benefit with HR between 0.52–0.58 with CDKi [16,19–21].

The present study was conducted to evaluate the real-world experience with cyclin dependent kinase inhibitors in metastatic breast cancer from India.

## Methods

This was an observational retro-prospective study carried out at Rajiv Gandhi Cancer Institute and Research Centre, New Delhi which is a tertiary cancer care centre. The study was conducted as per the Declaration of Helsinki and was given a waiver from the consenting process by the Institutional Review Board (Ethics Committee) of the Institute (vide letter no RGCIRC/ IRB-BHR 50/ 2021 dated 30/06/2021). The records were last accessed on 05/01/2024.

All patients with hormone receptor-positive Her2neu negative MBC who had received at least 3 months of CDKi in the first line (*de novo*) or subsequent lines after prior treatment (recurrent) setting with Eastern Cooperative Oncology Group (ECOG) Performance Status (PS)- 1,2,3 were included in the study. Patients with uncontrolled systemic dysfunction- hematological, hepatic, cardiac, renal, gastrointestinal, etc., unwilling to take CDKi or with prior exposure to CDKi were excluded from the study. Fitness for these drugs were assessed after routine laboratory tests (complete blood count, liver and kidney function tests including electrolytes), and cardiac evaluation (2-D Echocardiography and Electrocardiogram). All patients started on these CDKi were combined with endocrine therapy (along with ovarian suppression in premenopausal women). Palbociclib was started on a dose of 125 mg per day while Ribociclib was started on a dose of 600 mg per day- both were given for 21 days in 28-day cycles. Initial response assessment was done by PET-CT scan advised 3 months after starting the CDKi with follow up scans at 3−6 monthly intervals in 2nd-3rd years and 6 monthly thereafter. Suspicion of clinical disease progression was also checked with PET-CT scan. RECIST v 1.1 criteria supplanted with SUV max values were also used to document progression [22]. The response was categorized into the following types- Complete Response (CR), Partial Response (PR), Stable Disease (SD) and Progressive Disease (PD). CR, PR and SD were altogether considered for the calculation of clinical benefit rate.

Patients underwent routine laboratory investigations and also ECG on each visit. 2D Echo was repeated annually. Toxicity monitoring was reported as per CTCAE v 5.0 grades mentioned in FDA drug inserts of these drugs. The highest grade of toxicity witnessed during the entire period of treatment/ follow-up was registered. Dose reduction/interruption and/or discontinuation/restart were decided as per clinician’s discretion based on patient evaluation.

All statistical analyses were performed using MedCalc® version 20.115. All continuous variables were expressed as mean±standard deviation or median with interquartile range as per the distribution of data. Categorical variables were expressed as number and their respective percentages. Frequency distribution statistics were employed to analyze the epidemiological and treatment variables. Progression-free Free Survival was estimated using the Kaplan-Meier method and subgroup analyses were done employing two-sided log-rank test. Hazard ratios and confidence intervals (CIs) were estimated using a stratified Cox proportional hazards model. All these statistical results were accompanied by 95% confidence intervals (CI). p-value <0.05 indicated statistical significance.

## Results

Among a total of 143 patients, 93 and 50 patients had received palbociclib and ribociclib, respectively. The median age of the patients was 56 years (range 31–80 years, 95% CI 53–59 years). The most common factors in both the groups were age group 45–64 years (65.7%) and postmenopausal status (65.7%). Patients mostly belonged to ECOG Performance Status 1 (n = 66, 46.2%) and 2 (n = 59, 41.3%) groups. PS2 was the largest cohort (n = 40, 43%) in palbociclib group while PS1 was most commonly observed in the ribociclib group (n = 27, 54%). There was equivalent distribution among left and right-sided breast tumors in either group (50.5% and 48% right-sided tumors in palbociclib and ribociclib groups, respectively). There were more cases of *de novo* MBC (n = 83, 58.04%) as compared to recurrent MBC (n = 60, 41.95%). While more patients received palbociclib in the first line as compared to the second line, ribociclib was equally distributed among first and second lines (n = 24, 48%). Equivalent number of patients in the palbociclib group were chemo-naïve and exposed to prior one line of chemotherapy. On the other hand, ribociclib cohort had more patients with exposure to prior one line of chemotherapy as compared to chemotherapy- naïve patients. However, exposure to prior two lines of chemotherapy was more in the palbociclib group (n = 10, 10.7%) than in ribociclib (n = 1, 2%) group. It is important to mention here that prior chemotherapy includes chemotherapy received in neoadjuvant/adjuvant setting when the disease was not metastatic. Table 1 shows the demographic and clinical profile of patients with palbociclib and ribociclib.

**Table 1:** Demographic and clinical profile of patients with Palbociclib and Ribociclib N (%).

Characteristics	Palbociclib N (%)	Ribociclib N (%)	Total N (%)
Age			
<45	14 (15)	12 (24)	26 (18.18)
45-64	63 (67.8)	31 (62)	94 (65.73)
>65	16 (17.2)	7 (14)	23 (16.07)
Menopausal			
Pre	29 (31.2)	20 (40)	49 (34.26)
Post	64 (68.8)	30 (60)	94 (65.73)
ECOG PS			
1	39 (42)	27 (54)	66 (46.15)
2	40 (43)	19 (38)	59 (41.25)
3	14 (15)	4 (8)	18 (12.58)
Laterality			
Left	45 (48.4)	25 (50)	70 (48.95)
Right	47 (50.5)	24 (48)	71 (49.65)
Bilateral	1 (1.1)	1 (2)	2 (1.39)
Presentation			
*De novo*	56 (60.2)	27 (54)	83 (58.04)
Recurrent	37 (39.8)	23 (46)	60 (41.95)
Line of hormonal therapy			
First	40 (43)	24 (48)	64 (44.75)
Second	35 (37.6)	24 (48)	59 (41.25)
Third	18 (19.3)	2 (4)	20 (13.98)
>Four	0 (0)	0 (0)	0 (0)
Prior lines of chemotherapy			
None	40 (43)	16 (32)	56 (39.16)
One	43 (46.2)	33 (66)	76 (53.14)
Two	10 (10.7)	1 (2)	11 (7.69)
>Three	0 (0)	0 (0)	0 (0)
Initial Response			
Complete response	4 (4.3)	4 (8)	8 (5.59)
Partial response	60 (64.5)	32 (64)	92 (64.33)
Stable disease	19 (20.4)	10 (20)	29 (20.27)
Progressive disease	10 (10.7)	4 (8)	14 (9.79)

Anemia & transaminitis were the most common toxicities in both drug cohorts [palbociclib (97.8% & 75.3%, respectively) & ribociclib (96.5% & 82%, respectively)]. Table 2 shows the toxicity profile of patients with palbociclib and ribociclib. Overall, dose modification due to toxicity was not required in 67.8% patients. Dose reduction and dose interruption were required in 25 (17.5%) and 13 (9.1%) patients, respectively. Permanent dose discontinuation due to unmanageable recurrent toxicities was found in less than 5% patients. A total of 25 patients (17.49%) underwent dose reduction- 17 in palbociclib group and 8 in ribociclib group. Most patients underwent level I dose reduction; 13 patients (13.9% of 93) in palbociclib group and 7 patients (14% of 50) in ribociclib group.

**Table 2:** Toxicity profile of patients with Palbociclib and Ribociclib N (%).

Characteristics	Palbociclib N (%)		Ribociclib N (%)		Total N (%)	
**Any grade**	**Grade 3/4**	**Any grade**	**Grade 3/4**	**Any grade**	**Grade 3/4**
***Hematological***						
Neutropenia	86 (92.47)	21 (22.5)	50 (100)	12 (24)	136 (95.1)	33 (65.73)
Febrile neutropenia	14 (15.05)	4 (4.3)	10 (20)	4 (8)	24 (16.7)	8 (5.59)
Leukopenia	38 (40.86)	16 (17.20)	27 (54)	12 (24)	65 (45.45)	28 (19.58)
Anemia	91 (97.84)	26 (27.95)	47 (94)	7 (14)	138 (96.5)	33 (23.07)
Thrombocytopenia	71 (76.34)	14 (15.05)	16 (32)	4 (8)	87 (60.83)	18 (12.5)
***Non-hematological***						
Transaminitis	70 (75.26)	9 (9.67)	41 (82)	7 (14)	111 (77.62)	16 (11.18)
Mucositis	47 (50.5)	5 (5.37)	41 (82)	2 (4)	88 (61.53)	7 (4.89)
Diarrhea	57 (61.29)	3 (3.22)	38 (76)	1 (2)	95 (66.43)	4 (2.79)

**Fig 1:**
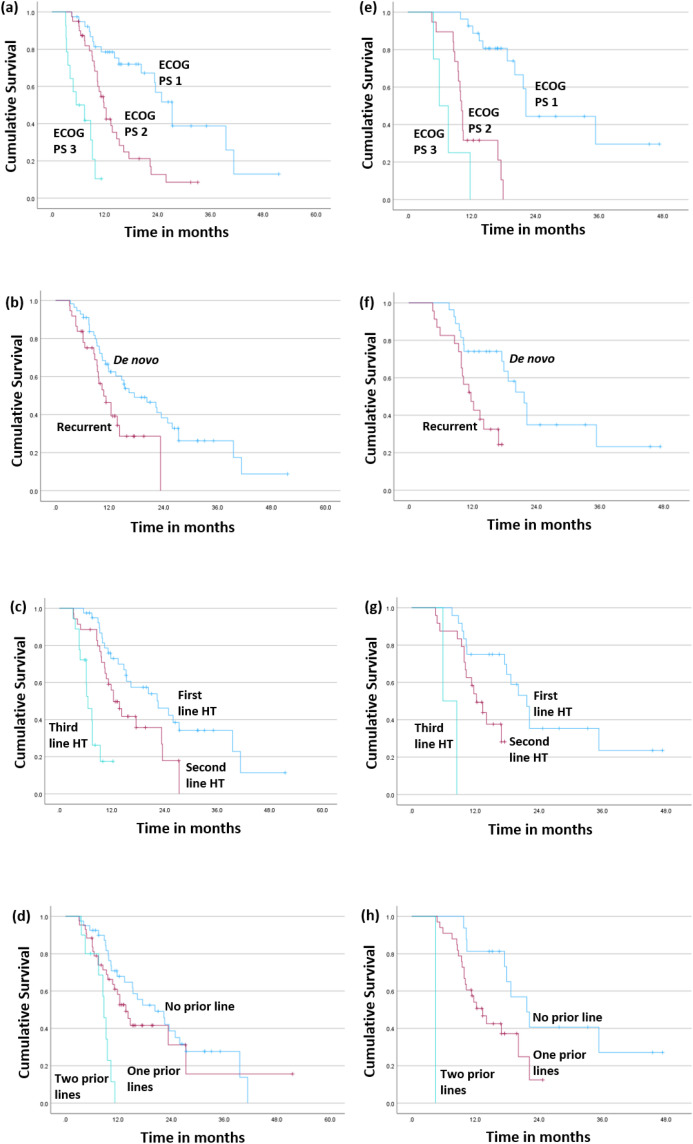
Progression free survival of patients receiving Palbociclib (a-d) and Ribociclib (e-h) with respect to Eastern Cooperative Oncology Group (ECOG) performance status (PS) (a,e); Disease at presentation (b,f); Line of hormonal therapy (c,g); Prior chemotherapy lines (d,h).

**Fig 2:**
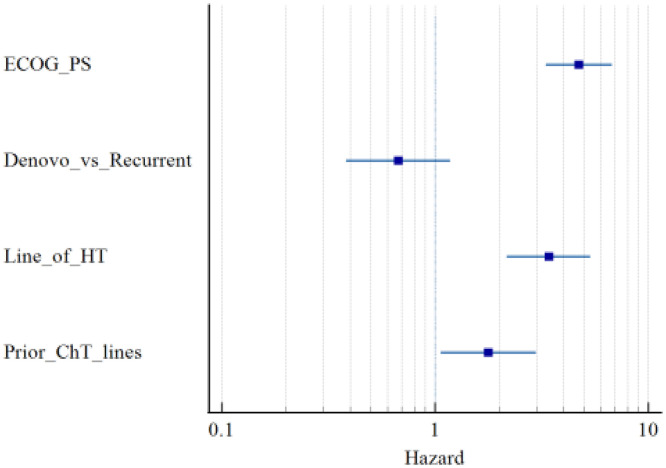
Forest plot with respect to various parameters.

The clinical benefit rate was 90.2%. The majority of the patients showed partial response (64.3%) followed by stable disease (20.3%) in either group. In the patients receiving palbociclib, a better median progression-free survival (PFS) was observed in patients with ECOG performance status (PS) 1 (27.3 months, p-value <0.0001), *de novo* disease (17.5 months, p-value 0.0155), first-line hormonal therapy (22.5 months, p-value <0.0001) and no prior chemotherapy (20.3 months, p-value 0.0001). Similarly, in the patients receiving ribociclib, a better median PFS was observed in patients with ECOG PS 1 (22.2 months, p-value <0.0001), *de novo* disease (21.7 months, p-value 0.0042), first line hormonal therapy 22.5 months (p-value <0.0001) and no prior chemotherapy (21.7 months, p-value <0.0001). On multivariate analysis, the factors significantly associated with PFS were ECOG PS, line of hormone therapy and prior chemotherapy (p-values <0.0001, < 0.0001 & 0.0269, respectively). Fig 1 shows the Progression free survival curves and Fig 2 shows the Forest plot with respect to various parameters.

## Discussion

The advent of CDKi has resulted in a paradigm shift in our approach to treat HR+ /Her2neu- MBC patients. The most common age group being 45–64 years corroborates with the previous study from our Institute by Agarwal *et al.* as well as with two other studies from India [23–25]. In fact, the median age of patients in PALOMA 3 trial was also the same [9]. Ribociclib cohort patients had a median age of 53.5 years which is younger than patients enrolled in MONALEESA 2 and 3 (median age 62–63 years) [26–27]. The median age was similar to that of Australian patients (54.3 years) but this population was younger as compared to the European (61–65 years) and American patients (66 years) [28–31]. This reflects a younger age of presentation of MBC in India as confirmed by the age-specific incidence rates across the world [32].

Most patients were postmenopausal (n = 94, 65.7%) akin to the predecessor studies from India [23,33]. It follows suit to other patient population from other geographical areas like Australia 76%, Europe 80.6%, and US 85.5% [28,31,34]. Patients mostly belonged to ECOG Performance Status 1 (46.2%) and 2 (41.3%) in either of the CDKi group, similar to the study by Rath *et al.* [33]. Real world data from France, Australia and US also mimics this pattern [28,35–37]. Although there was no significant difference in ECOG PS between the two groups (Chi square test p = 0.285), proportion of ECOG PS 3 was lesser in the ribociclib group (8%) as compared to palbociclib group (15%). This again reflects the preference of palbociclib over ribociclib in poorer PS patients, possibly due to greater experience and confidence of clinicians with palbociclib. The majority of our patients had *de novo* MBC (58%) as compared to recurrent (42%) disease in both the CDKi groups. The RWD from the US by Law *et al.* had similar findings while other real world data from the US and UK showed a reverse pattern [2,5,9,12]. MONALEESA-3 and 7 had 98−100% patients with metastatic disease at study entry while MONALEESA-2 had more recurrent disease than *de novo* MBC [26,38,39]. PALOMA- 2 study among Asian population had only 20% patients with stage 4 disease at initial diagnosis [40]. The discordance is due to different patient inclusion criteria and geographical variations across these studies. Majority of patients in either cohort (82.7% in palbociclib & 86% in ribociclib) had visceral disease as opposed to bone-only metastatic sites. A similar pattern of >80% cases having visceral disease was noticed in both the previous Indian studies [23,33]. A greater prevalence of visceral disease has been noted in RWD studies from USA, Canada, and Australia although not so disproportionate as compared to the Indian studies [34,36,41]. This might be one of the reasons why Indian MBC patients have inferior survival as compared to their western counterparts.

With respect to the line of starting CDKi, patients were equally divided among first and second lines in ribociclib group (n = 24, 48% in both 1st line and 2nd line) while greater preponderance was noticed in the first line (43%) over the second line (37%) in palbociclib group. Equivalent distribution of CDKi in the first and second lines (21.8% and 24.8%) was noticed in another study from India [33]. European studies by Fountzilas *et al.* and Porte *et al.* have also shown a greater prevalence of CDKi in the first line as compared to the second line [31,35]. Most patients had received prior one line of chemotherapy (including neoadjuvant/adjuvant treatment) in our study (46.2% in palbociclib group, 66% in ribociclib group). The previous institutional experience in 50.5% patients also affirms the present finding [23].

The clinical benefit rate was 90.2%. The most common response on PET-CT scan after at least 3 cycles of the drugs was partial response (PR) (n = 92, 64.3%) followed by stable disease (SD) (20.3%) and progressive disease (PD) (n = 14, 9.8%). A similar trend was documented by previous two Indian studies [23,33]. While MONALEESA- 2 corroborated with this pattern, MONALEESA-3 and PALOMA-3 had more SD as compared to PR [26,27,42]. The systematic review by Harbeck *et al.* on RWD also noted SD as the most common overall response to CDKi [25]. This discordance can be explained by better sensitivity of response documentation by PET-CT as used in our Institute in contrast to CT/MRI used in the landmark trials.

Most patients (62.9%) had disease progression in either of the CDKi groups. The median progression-free survival (mPFS) was 14.2 months (95% CI 11.1–22.3) and 17.5 months (95% CI 11.3–21.7) for palbociclib and ribociclib, respectively (log-rank test p = 0.498). Poor ECOG PS conferred poorer mPFS in both drug cohorts. mPFS estimates were 27.3, 11.8 and 5.5 months (log-rank p < 0.0001) in the palbociclib cohort and 22.2, 10.1 and 5.9 months (log-rank p < 0.0001) in the ribociclib cohort for ECOG PS 1, 2 and 3 respectively. Cox regression multivariate analysis also confirmed significant influence of ECOG PS on PFS- HR [4.728 (95%CI 3.31–6.74)] [33]. Table 3 shows the real world studies on cyclin dependent kinase inhibitors with respect to progression free survival. Real-world data have also shown inferior survivals with ECOG PS 2,3 as compared to ECOG PS 0,1 highlighting the impact of ECOG PS > 2 which is usually excluded from the clinical trials [28,30,33,35–37,43]. *De novo* metastatic disease had significantly better mPFS as compared to recurrent disease in both palbociclib group (17.5 *vs.* 10.7 months, log rank p = 0.0155) and ribociclib group (21.7 *vs.* 12.2 months, log-rank p = 0.0042). A previous study from our Institute also noted a 3-year PFS of 86% *vs.* 64–68% for *de novo vs.* recurrent MBC (p = 0.108) [24]. DeMichelle *et al.* reported the highest PFS benefit with palbociclib + letrozole for a gap between diagnosis to metastatic disease as less than a year as compared to gaps of more than a year or *de novo* metastatic disease: PFS HR 0.39 *vs.* 0.57–0.61 and OS HR 0.22 *vs.* 0.56–0.78 [36]. Rugo HS *et al.* similarly showed PFS HR 0.65 *vs.* 0.68–0.87 with respect to a gap of <1 year *vs. de novo* or >1 year [37]. Law *et al.* have also echoed this finding: mPFS 13.3 months *vs.* 31.6 months for DFI (diagnosis in early stage to metastatic disease) 1 year respectively [28]. Multivariate analysis by DeSouza *et al.* identified the presence of *de novo* MBC as a significant factor for improvement in PFS (HR 0.56, 95%CI 0.37–0.85, p = 0.006) [44]. Irrespective of the CDKi used, there was a significant difference in mPFS- 22.2 (95%CI 16.3–27.3), 13.4 (95%CI 10.7–17.5) and 6.5 months (95%CI 4.7–8.5) in first, second and third therapy usage, respectively. Cox regression multivariate analysis reaffirmed the significant impact of the line of HT on PFS- HR 3.411 (95%CI 2.17–5.36), p < 0.0001. A previous institutional study by Agarwal *et al.* showed mPFS of 20 and 12 months with usage of palbociclib in the first and second lines, respectively [24]. Slightly better mPFS in the present study might be due to the inclusion of retrospective patients on follow-up who had a better PFS as compared to those who could not make up for follow up within the study period. Corresponding figures for CDKi in the first line and >2 lines in another study from India were 21.1 months [33]. The European study by Fountzilas *et al.* also showed significant benefit in mPFS - 18.7, 12 and 7.4 months [31]. Real-world data from the USA, Australia, and Germany also correlate with the same trend in PFS with respect to prior lines of endocrine therapy [30,34,45]. PFS significantly decreased with the number of previous chemotherapy lines, none *vs.* one line *vs.* two lines in both palbociclib (20.3 *vs.* 13.7 *vs.* 8.7 months, log-rank p = 0.0001) and ribociclib cohort (21.7 *vs.* 14.1 *vs.* 4.5 months, log-rank p < 0.0001). A significant impact of prior chemotherapy lines on PFS was also confirmed on Cox regression multivariate analysis- HR 1.744 (HR 1.067–2.948), p = 0.0269. However, Rath *et al.* did not notice any significant improvement in PFS with absence of prior chemotherapy – HR 0.631 (95%CI 0.333–1.194, p = 0.157) [33]. Schettini *et al.* in their meta-analysis on PALOMA, MONALEESA and MONARCH trials noted better pooled OS HR of 0.72 (95%CI 0.55–0.93, pooled p = 0.01) as compared to 0.85 (95%CI 0.61–1.18, pooled p = 0.34) with CDKi against endocrine therapy alone or no prior chemotherapy *vs.* prior chemotherapy, respectively in metastatic setting [16].

**Table 3:** Real world studies on cyclin dependendent kinase inhibitors with respect to progression free survival.

References	Median Progression free survival (mPFS)
Agrawal *et al.* [23]	20.2 months (first line) *vs*. 12 months (second line)
Fountzilas *et al.* [30]	18.7 months (first line) *vs*. 12 months (second line)
Rath *et al.* [32]	21.1 months
Wong *et al.* [33]	Not reached
DeMichele *et al.* [35]	19.7 months
DeSouza *et al.* [43]	21.3 months (first line) *vs*. 12.2 months (second line)
Knudsen *et al.* [44]	27.6 months (letrozole) *vs*. 17.2 months (fulvestrant)
Present study	15.2 months

Hematological toxicities were predominantly neutropenia and anemia experienced by 86 (92.4%) and 91 (97.8%) patients in palbociclib group and 50 (100%) and 47 (94%) patients in ribociclib group, respectively. Febrile neutropenia was experienced by 14 (15.05%) and 10 (20%) patients in palbociclib and ribociclib cohorts, respectively. Palbociclib in combination with letrozole and fulvestrant resulted in all-grade neutropenia in 80% patients, while Grade 3–4 neutropenia was observed in 66% and 65% patients, respectively in the PALOMA trials [46]. In the case of ribociclib, combination with letrozole and fulvestrant resulted in all grades of toxicities in 75% and 70% patients, while grade 3–4 neutropenia was experienced by 59% and 53% patients, respectively in the MONALEESA trials [46]. In addition, febrile neutropenia was seen with ribociclib in 5 patients (1.5%) in MONALEESA-2 trial and 7 patients (2.1%) in MONALEESA-7 trials [46]. Although rates of neutropenia (all grades and Grade 3–4) corroborate with our findings, we had more frequencies of febrile neutropenia, probably due to the inclusion of ECOG PS 2–3 and elderly (>75 years) patients who were often heavily pre-treated. It is noteworthy that any-grade of neutropenia, leukopenia, and febrile neutropenia as well as all non-hematological toxicities had higher frequencies in the ribociclib cohort, similar to that observed in the TMH study [33].

Breast cancer treatment has greatly improved with the employment of nanoparticle-based systems, including liposomes, nanostructured lipid carriers, polymeric micelles, polymeric nanoparticles, dendrimers, and hybrid nanoparticles. Nanomedicine can improve the drug efficacy by better targeting the tumor with more deeper tissue penetration [47]. This can hold true for the newer targeted therapies being used in the treatment of breast cancers. Also, research involving genomics and proteomics in this area may also pave the way for novel biomarkers in order to improve the survival rates in breast cancer patients [48].

There are, however, a few inherent limitations of the study including its retrospective nature. The sample size of the study is small and the groups are heterogenous in their characteristics. Initial response assessment was done by PET-CT scan advised 3 months after starting the CDKi and hence patients who progressed or died before 3 months were excluded which may have affected the response/ survival rates. However, this is one of the largest studies in the Indian population sharing the experience with the cyclin dependent kinase inhibitors in metastatic breast cancer and comparing the effectiveness of palbociclib vs. ribociclib was not the aim of the study. Nevertheless, the biggest strength of the study lies in the survival data which is the most important end-point in the study.

Overall, the present study shows the real-world experience with CDK inhibitors in metastatic breast cancer in India. However, large-scale multi-centric studies are required to evaluate the nationwide Indian experience with these inhibitors.
